# Life dispositions of caregivers of a family member with mental disorder

**DOI:** 10.3389/fpsyt.2025.1496329

**Published:** 2025-09-09

**Authors:** Elena Mihaylova Ivanova, Margarita Angelova Stefanova-Bakracheva, Vihra Krumova Milanova

**Affiliations:** ^1^ Alexandrovska University Hospital, Clinic of Psychiatry, Faculty of Medicine, Medical University, Sofia, Bulgaria; ^2^ Faculty of Educational Studies and the Arts, St. Kliment Ohridski Sofia University, Sofia, Bulgaria; ^3^ Alexandrovska University Hospital, Clinic of Psychiatry, Sofia, Bulgaria

**Keywords:** well-being, meaning in life, self-esteem, coping strategies, caregivers of a family member with mental disorder

## Abstract

**Background:**

Despite the shift in the focus observed recently on supporting the family rather than only providing care for its member with a mental disorder, there are still many problems faced by the caregivers leading to dysfunction in families with such a member, both as on family and on individual level.

**Aim:**

The aim of the present study was to identify similarities and differences in the adjustment pathways of caregivers of a parent or child with mental disorder, as compared to a control group, as well as to determine the specific life dispositions of parents caring for child with mental disorder and of children caring for parent with mental disorder.

**Method:**

The design of the study is cross-sectional, comprising a convenience sample of 167 respondents: 82 parents and 46 children caregivers and 39 respondents in the control group, who were administered eight scales, measuring their life orientation, well-being, meaning in life, preferred coping strategies, self-esteem, social anxiety, and depression.

**Results:**

Significant differences were reported between the control group and the groups of caregivers in respect to well-being and self-esteem, which were higher among the respondents from the control group. Indicative result was the lack of difference in the level of depression between the control group and the caregivers, revealing the result of self-regulation and the different pathways, leading to it. While parents caring for a child with mental disorder had higher orientation children caring for a parent with a mental disorder appeared to be the most vulnerable group – they were avoidant oriented, had lowest well-being, self-esteem and meaning in life and optimism, lacked positive emotions, reported unsatisfactory relationships, lower engagement, and were in ongoing search for meaning in life, and experienced of loneliness to the greatest extent.

**Conclusions:**

The specific adjustment profiles of parents and children caregivers highlighted their different needs for support. While parents may benefit from informational support and guidance, children may benefit more from motivational support to maintain meaning in life. The outlined individual differences may also contribute to family support and counselling aimed at improving functioning of the family system and the performance of its individual members.

## 1 Introduction

Mental disorders are becoming increasingly common, with some researchers reporting that approximately 19% of the world’s population has at least one mental disorder (1). Mental disorders are socially significant diseases, often chronic, resulting in disability and high cost to society. These illnesses also affect the lives of the whole family that faces the challenge of adapting to the situation. Over the years, there has been a shift from prioritizing inpatient care for people with mental disorders to caring for them in family and community settings. Family-based care supports the patient’s recovery and return to activities and work. The trend towards family-based care (2) makes the family the primary support system that provides sustainable care for people with mental disorders (3). Family care takes place in close proximity to the health care system and is therefore seen as an “institution” in which patients with mental disorders live (4). In addition to supporting the patient, family care supports the therapeutic process and thus helps to achieve control over the symptoms of the illness (5).

By taking on responsibility for the patient, however, family members are exposed to additional psychological distress and social consequences that affect the functioning of the whole family (6, 7). The responsibilities and strains on the family begin at the onset of the mental disorder and continue throughout the patient’s life (4). Caregivers are challenged by the difficulty of gaining control over the disease symptomatology, the ongoing demands and obligations associated with mental illness, and the negative societal attitudes (8). In the process of caring, family members are exposed to tremendous stress and negative emotional experiences, creating risks for their mental health and social functioning. According to researchers, the constant need for adaptation creates real difficulties for the caregivers and thus providing care affects their lives (3). The family members of a person with a mental disorder are even referred to as ‘second-tier patients’ (2), as they take on a large part of the responsibility of caring for the mentally-ill person and this care can worsen the caregiver’s quality of life (5), with the risk of creating a vicious circle (9) leading to deterioration and relapse of the mental disorder in the patient, who may be their child or parent.

Caring for a mental patient becomes a heavy burden for family members, as they have to take on many of the caring responsibilities and fulfill different roles (10, 11) in order to meet the cost of the patient’s treatment, which is an additional financial problem for them (12, 13). Caring for a mental patient has an impact on the social functioning of family members, most commonly expressed in distancing/isolation from friends and acquaintances (13, 14), difficulties in their studies and career (15), difficulties in achieving a work-life balance (10, 16, 17); they also have to cope with the negative attitudes of others towards families with a mental patient (18). Social isolation most often affects the patient and the caregiver (19, 20), but the mental disorder is most often a family secret and its disclosure is accompanied by a significant conflict (21). Caregivers of mental patients report feelings of helplessness (3, 19, 20), shame and embarrassment (11, 19– 20), anger, fear, anxiety and depressive experiences (3, 11, 19, 22), monotony in their lives largely due to caring (11, 14), lack of security (11), low self-esteem (20), frustration caused by the chronic nature of the mental disorder, feelings of guilt, self-blame or blaming the patient (3, 19, 20), worries about the patient’s future (18) and worries about their own future (11, 18). Manifestations of distress are associated with different roles in the family, including role reversal, often reinforced by the efforts to keep the mental disorder secret (20).

A number of researchers have pointed out that caregivers of a person with a mental disorder need information about the symptoms and course of the mental disorder (12), proven treatment modalities (23), recognition of early warning signs of worsening of the mental disorder (8), changes in lifestyle (individual, social and work opportunities) due to the mental disorder (22), and ways of communicating with the patients to avoid and prevent conflicts with them (10), which would help caregivers to adapt to their role (24).

Research has been done on the implications of a parent’s mental disorder on child development and of a child’s mental disorder on his/her parents. With regard to children of a mentally ill parent, research indicates that globally between 15 and 23% of children live with a mentally ill parent (25) and that they are at 5.2 times higher risk of depression and 3.7 times more vulnerable to anxiety disorder compared to their peers with healthy parents (26). Parenting styles and the parent-child relationship have a crucial impact on the child’s development, personality formation and mental health (27). Children raised by a parent with a mental disorder face a number of risk factors that affect their development: family dysfunction, high levels of stress, experiences of guilt, shame and loneliness, lack of social acceptance and social support. Because of the mental disorder, the parent may have difficulty fulfilling his or her responsibilities, leading to parentification - the child becomes the parent and cares for his or her ill mother or father (28). Children with a mentally ill parent have to cope with additional risks resulting from the lack of social support and poverty (29).

Several authors have pointed out that parents of children with ASD have more pronounced manifestations of social anxiety, while self-esteem and life satisfaction are much lower. Family members perceive the diagnosis of a mental disorder in a child as a stressful event leading to a crisis in the family (30), as confirmed by studies on families with a child with autism (31) and families with a child with intellectual disability (32). Social anxiety is more marked in parents of children with ASD (33). Caring for these children is associated with significant difficulties, parental conflict, worsening family dynamics, with the greater proportion of the ‘burden’ borne by mothers (34), as care and compassion are entrusted to women (35). A number of studies have highlighted the mental health risks and the reduced quality of life in caregivers of mentally ill people, the predominant part of which are women (36). Survey data have shown that more than half of the caregivers of mentally ill people are women (37). Other authors have also confirmed this trend of women bearing the greatest burden of care. Researchers have pointed out the need of improved awareness of mental illness; the presence of social stigma; the deterioration of relationships; the blame that relatives receive for having a family member with a mental disorder (38) and that this refers especially to women, representing more than half of the caregivers (39).

Increasingly, researchers have been pointing to the need for psychological education and rehabilitation programs that involve both the person with a mental disorder and the caregiver - a combined approach, rather than focusing solely on the person with a mental disorder (40). The support may involve instrumental support (help with daily needs and household activities), emotional support, and knowledge acquisition (information from professionals, for example, and exchange of experiences with other caregivers of people with mental disorders) (41). Mental disorders can be chronic and debilitating, requiring caregivers to care for patients throughout their lives. Long-term care and the assumption of multiple responsibilities have led authors to suggest that mental disorders are devastating for both the patient and the caregiver (42).

## 2 Materials and methods

### 2.1 Research aim

Given the three main research lines in the references, eg. 1) the difficult setting of caregivers, 2) the need of information and support, and 3) the clearly highlighted need of work with families, little is known about the specific needs and profiles of the caregivers by the means of comparing their life dispositions. The aim of the study was to contribute and produce preliminary findings concerning the well-being and its components, life orientation and coping strategies, self-esteem, social anxiety and depression in parents who have a child with a mental disorder and in children (over 18) caring for a parent with a mental disorder, as well as to compare their results depending on the caregiver’s position with those of the control group - healthy parents of healthy children whose parents are also healthy. The specific focus of the study was on analysis of the different pathways in the process of adaptation in view to the outlined disadvantages faced by caregivers of family member with mental disorder, eg. lower self-esteem, higher level of anxiety and depression, reduced quality of life and well-being and the specific outcomes for parents of child with mental disorder and children of mentally ill parents (3, 10–33). Тhis led to three hypotheses:

H1: The control group is expected to have more optimistic life orientation, higher levels of well-being and experienced positive emotions and happiness, higher self-esteem and lower levels of depression, preference of proactive coping over avoidance coping and learned helplessness compared to the two groups of caregivers. Furthermore, significant differences are expected to be outlined regarding the specific patterns in life disposition and preferred coping between parents and children caregivers.

H2: The self-esteem as a result of perceived life adjustment is expected to have different independent predictors for the control group and the two groups of caregivers.

H3: The individual variables gender, age, marital status and education level are expected to have a partial but significant effect for the control group and caregivers well-being and life dispositions.

### 2.2 Organization of the study

All respondents volunteered to be involved in the study. The data for the control group and caregivers of family patient with mental disorder were collected in the period June 2023 - June 2024 by a psychiatrist and a child psychiatrist and a general practitioner. Inclusion criteria for all respondents caregivers was analysis of the medical records of the family member with mental disorder and ICD-11 diagnosis, with the requirement of more than one year elapsing since diagnosis and ongoing maintenance medication, with no change in the therapy for the last 6 months. Inclusion criteria for the control group (healthy parent and child, no evidence of mental disorder whose parents are healthy) involved screening with D. Goldberg’s General Health Status Questionnaire (GHSQ) (43), adapted for the Bulgarian population with a normative threshold of ≤ 11 points (44). All healthy controls included in the study were within the defined norm for the Bulgarian population.

An informed consent was obtained from all respondents, who were aware of the aim of the study and that aggregated results will be included in open access publications. The results were anonymоus and no identifying data were included in the protocols during the data collection. Participants were informed also that the participation is voluntary, that they can withdraw at any time, and that the information collected is confidential. Informed consent and instruments were administered using paper-and-pencil method. The instruments had only thick boxes and after completion were collected in a box in view to guaranteed anonymity of the responses. The informed consent was signed, as respondents were instructed to put initials at their choice, not revealing their real name and surname and collected separately from the instruments in a different box.

### 2.3 Instruments

All 167 respondents completed a questionnaire consisting of 151 items, comprising eight scales, measuring their life orientation, well-being, meaning in life, self-esteem, coping strategies, learned helplessness, social anxiety and depression. For all scales, a 5-item self- report Likert-type scale (strongly disagree, disagree, neither disagree nor agree, agree, strongly agree) was used. Only the depression scale had a 4-point rating scale (never/rarely, sometimes, often, very often/all the time). Twenty-four variables were studied as expressed in the three groups of respondents: control group, caregivers of a child with mental disorder, and caregivers of a parent with mental disorder: *Life orientation* (optimism/pessimism), *PERMA Profiler* (positive emotions, negative emotions, engagement, relationships, meaning, achievements, assessment of physical health, happiness, loneliness), *Meaning in life and Search for meaning, Coping* (proactive coping, reflective coping, strategic planning, preventive coping, seeking instrumental support, seeking emotional support, avoidance), *Learned helplessness, Self-esteem, Social anxiety, Depression*. Individual variables included gender, age, education level, marital status. Administered eight scales and reliability coefficients are given below. All scales had been previously piloted after three translations and one back translation and revealed good psychometric results.

1) For measurement of self-esteem was administered the *Rosenberg’s Self-Esteem Scale* (45), comprising 10 items (α = .789). 2) Optimism *vs*. pessimism as life orientation was measured with the 10-items *Life Orientation Scale* (46) (α = .729 for optimism and α = .698 for pessimism). 3) The *Meaning in Life Questionnaire (MLQ)* (47) was used to measure meaning in life or search of meaning in life. The scale comprises 10 items, 5 for each variable (α = .812 for meaning in life and α = .897 for search for meaning). 4) In terms of coping, the *Proactive Coping Inventory (PCI)*, a multidimensional research instrument (48), was used to assess the expression of proactive coping, reflexive coping, strategic planning, preventive coping, seeking instrumental support, seeking emotional support and avoidance. The scales used were as follows: 14-item proactive coping scale (α = .874); 11-item reflexive coping scale (α = .863); 4-item strategic planning scale (α =.840); 10-item preventive coping scale (α = .816); 8-item instrumental support seeking scale (α = .884); 5-item emotional support seeking scale (α = .756); and 3-item avoidance scale (α = .820). 5) The *Learned Helplessness Scale* (49) was administered to measure the perceived helplessness. The scale is unidimensional and comprises 20 items (α = .949). 6) For measurement of well-being was administered the 22-items *PERMA Profiler* (50), comprising items assessing positive emotions, engagement, relationships, meaning in life, and achievement (3 items for each of the 5 variables: positive emotions (α = 757), engagement (α = .689), relationships (α = .753), meaning in life (α = .781), and achievement (α = .723); and in addition 8 items, measuring the overall happiness, loneliness, negative emotions and health. 7) The *State Social Anxiety Scale (SSA)* (51) determines social anxiety and contains 8 items (α = .903). 8) The 20-item *Self-Rating Depression Scale* (52) was used to assess depression (α = .797).

Data were processed using IBM SPSS Statistics 25. Effect size was calculated with the e-calculator, available at https://lbecker.uccs.edu/. Descriptive statistics, reliability tests using Cronbach’s alpha and item analysis, principal components analysis with rotation, Kolmogorov-Smirnov test, ANOVA, t-test, correlation analysis, and regression analysis, were employed for data processing. Effect size was measured both as between group difference and as strength of the relationship.

A preliminary screening of the data for assumptions of univariate normality was performed with determination of skewness and kurtosis of distribution. All observed indices were below the acceptable threshold for excess and kurtosis (± 2). Screening for outliers was also performed. Standard rules were followed for component analysis and reliability. For all scales Cattell’s scree plot and an exploration analysis by principal components method with Varimax rotation was performed. Followed rules was Kaiser-Meier-Olkin (KMO) test for overall sample adequacy to have KMO value >.6; the result of the Bartlett’s test of sphericity to test correlations between variables to be valid (accepted criterion for significance is p <.01); the generated factor model to explain 50% of the total variance, given extracted factors with eigenvalue > 1.0 (Kaiser normalization criterion) and given the sample size, the value of the factor weight should be >.4 (given the conservative criterion of including in the pattern-matrix only items with factor weights of.6 and values depending on the sample size). In terms of reliability the value of Cronbach’s alpha to be >.70 (with adjustment for brief scales of.60); the correlation between individual items and the whole scale should be greater than.400 (using the Spearman-Brown prediction formula). Stepwise regression was used for the regression model, starting with the strongest predictor and adding additional predictors that explained a significant amount of additional variance for the criterion, with an inclusion criterion of *p = .*1. All regression analyses had 95% confidence intervals, collinearity and pre-screening for outliers.

### 2.4 Sample

The respondents were 167, divided into three groups: 82 parents (49.10%) caring for child with a mental disorder, 46 children (over 18 years of age) - 27.50% caring for a parent with a mental disorder and 39 healthy parents (23.40%) of healthy children who had also healthy parents (control group). The distribution of all participants according to their education, marital status, gender, mean age, and mental health, is presented in **Table 1**.

**Table 1:** Sociodemographic data of the three groups.

Respondents Variable		Control group N=39		Parents caregivers N=82		Children caregivers N=46	
		N	%	N	%	N	%
Education	Higher	23	58.97	47	57.32	27	56.70
	College	0	0.00	1	1.22	4	8.70
	Secondary	16	41.03	30	36.59	13	28.26
	Primary	0	0.00	4	4.88	2	4.35
Marital status	Married	24	61.54	46	56.10	15	32.61
	Single	0	0.00	0	0.00	21	45.65
	Divorced	1	2.56	11	13.41	3	6.52
	Widow	0	0.00	2	2.44	0	0.00
	Single mother	4	10.56	8	9.76	2	4.35
	Cohabitation	10	25.64	0	0.00	4	8.70
Gender	women	33	85.00	70	85.00	36	80.00
	men	6	15.00	12	15.00	9	20.00
Mean age - years		38.77 ± 7.08		41.39 ± 7.21		33.29 ± 8.69	
Mental health - caregivers				20 mothers with a mental disorder (13 depression, 7 panic disorder)		12 children with mental disorder (10 with depression and 2 with panic disorder)	
Family anamnesis		N=40 have N = 87 have no family anamnesis					

The respondents with higher education predominated in the three groups studied - 97 (58.10%). Among the married people, largest was the number of parents caring for children with mental disorder - 46 (56,10%), whereas in the children caring for their mentally ill parents the number of single people predominated – 21 (45,65%). The mean age of all respondents was 38.57 ± 8.29 years, with the youngest being 18 years and the oldest 56 years. According to age, the participants were distributed into the following groups: under 30 - 33 (19.60%), between 30 and 40 - 56 (33.3%), between 40 and 50 - 69 respondents (41.35%) and over 50 - 9 respondents (5.4%). In all groups the female caregivers were larger in number than males. The distribution of the women in the three groups under study was the following: 33 healthy controls (84.62%), 70 mothers (85.37%) of a child with a mental disorder and 36 daughters (80.00%) caring for a parent with a mental disorder. The mean age of all women in the study was 38.43 ± 7.90 years. Youngest were the daughters caregivers - 33.25 ± 8.44 years, followed by the mothers in the control group - 38.72 ± 6.70 years and oldest were the mothers of children with mental disorders - 40.96 ± 6.88 years. This confirmed the data reported to date (35–39). The highest number of parents with two children was 73 (43.70%), followed by those with one child - 63 (37.70%), and 3 families had twins - 3.7%. The mean age of the children was 9.99 ± 4.42 years, with the youngest child being 4 years old and the oldest being 17 years old. Out of the respondents without children, 27 (16.20%) belonged to the group of children caring for a mentally ill parent. The distribution of children diagnosed with mental disorders in parental care was as follows: 34 with intellectual disability (20.30%), 34 with autism (20.20%) and 14 with schizophrenia (8.30%). The first symptoms of the mental disorder had been noticed by the parents at a mean age of 4.44 ± 3.31 years, with a period of two to three years between the onset of symptoms and establishing the diagnosis. 20 mothers with a mental disorder were included in the group of parents caring for mentally ill children: 13 mothers had depression (7 of which had postpartum depression that triggered off depression later in life) and 7 mothers had panic disorder. Their average age was 39.9 years, with an average age of giving birth 33.14 years. As a whole, the average age of parents caring for mentally ill children in the present study was the highest, as compared to the other two groups studied, reflecting the current trend of delaying parenthood. Women frequently have aspirations for higher education, so they become mothers and take on different social roles and responsibilities with regard to others at a slightly later age. 12 children with mental disorder (10 with depression and 2 with panic disorder) were caring for 10 mothers and 2 fathers with a mental disorder. The average age at which their parents were diagnosed with the mental disorder was 25.25 years. For the diagnosis of the parents the distribution is paranoid schizophrenia 14 (17%), ASD 34 (41%), mental retardation 28 (34%) and moderate mental retardation 6 (7%). 32 of the parents are mothers (67%) and 13 (28%) fathers. Position of children caregivers of a mentally ill parent is: first child – 10, second/third child – 10; only child – 26.

## 3 Results

### 3.1 General description

As can be seen in **Table 2**, pessimism, avoidance, social anxiety and learned helplessness were on the average below the theoretical mean of the scale. This also held true for self-esteem, which is due to data aggregation - self-esteem in the control group had a mean value higher than the theoretical mean of the scale (M = 3.17). Negative emotions also averaged above the theoretical mean of the scale, as did the absence of experienced loneliness. The remaining means, as expected (H1), added to the favorable overall picture observed in the entire sample, not only in the control group: higher values were obtained for optimism than pessimism (t = 10.983; p = .000; df = 166), positive than negative emotions (t = 5.242; p = .000; df = 166), engagement, relationships, meaning in life, happiness, presence of meaning in life, physical health assessment. All respondents revealed slightly higher mean values for effective coping, proactive coping, preventive coping, strategic planning, seeking instrumental and emotional support, and reflective coping. Depression was slightly above the theoretical mean of the scale. Variable variance was medium to high due to self-assessment.

**Table 2:** Descriptive statistics for all variables in the whole sample.

	Minimum	Maximum	Mean	Std. deviation	Variance	Skewness		Kurtosis	
	Statistics	Statistics	Statistics	Statistics	Statistics	Statistics	Std. error	Statistic	Std. error
Life orientation									
1 optimism	1.00	5.00	3.62	.845	.714	-.806	.188	.785	.374
2 pessimism	1.00	5.00	2.43	.851	.724	.462	.188	-.055	.374
PERMA profiler									
3 positive emotions	1.00	5.00	3.54	.704	.496	-.640	.188	.694	.374
4 negative emotions	1.67	4.67	3.09	.595	.354	.172	.188	.300	.374
5 engagement	2.00	5.00	3.63	.611	.374	-.207	.188	-.098	.374
6 relationships	1.33	5.00	3.71	.772	.596	-.415	.188	-.016	.374
7 meaning in life	1.00	5.00	3.96	.755	.571	-.917	.188	1.231	.374
8 achievements	2.00	5.00	3.74	.559	.312	-.278	.188	.205	.374
9 physical health assessment	1.00	5.00	3.68	.919	.844	-.457	.188	-.141	.374
10 happiness	1.00	5.00	3.48	.798	.637	-.363	.188	.268	.374
11 loneliness	1.00	5.00	3.52	.863	.745	-.189	.188	-.351	.374
12 Meaning in life	1.40	5.00	3.75	.691	.476	-.460	.188	.380	.374
13 Search for meaning in life	1.00	5.00	3.08	.938	.880	-.298	.188	-.880	.374
Coping									
14 proactive coping	1.54	5.00	3.65	.587	.345	-.478	.188	1.083	.374
15 reflexive coping	1.45	4.82	3.51	.636	.405	-.652	.188	.755	.374
16 strategic planning	1.00	5.00	3.30	.881	.775	-.188	.188	-.056	.374
17 preventive coping	1.50	5.00	3.41	.633	.401	.173	.188	.502	.374
18 search for instrumental support	1.00	5.00	3.36	.729	.532	-.208	.188	.265	.374
19 search for emotional support	1.00	5.00	3.38	.752	.566	-.190	.188	.568	.374
20 avoidance	1.00	5.00	2.18	.836	.700	.562	.188	.548	.374
21 Learned helplessness	1.00	4.95	2.36	.687	.472	.737	.188	1.460	.374
22 Self-esteem	2.30	3.80	2.99	.284	.081	.441	.188	.224	.374
23 Social Anxiety	1.00	5.00	2.52	1.01	1.015	.420	.188	.601	.374
24 Depression	1.30	3.25	2.11	.401	.161	.328	.188	-.302	.374

### 3.2 Comparison between groups

The hypotheses that the results of the control group and the groups of caregivers will differ (H1) was confirmed as different pathways, outlining self-regulation and life dispositions had been reported between the control group and the two groups of caregivers, as well as between the parents and children caregivers. Significant between-group differences were observed in optimism (F = 3.069; p = .049); positive emotions (F = 5.981; p = .003); (F = 2.822; p = .041); negative emotions (F = 4.203; p = .017); engagement (F = 24.510; p = .012); relationships (F = 3.182; p = .044); meaning in life (F = 3.866; p = .023); achievements (F = 8.483; p = .000); physical health assessment (F = 3.562; p = .014); happiness (F = 4.96; p = .008); loneliness (F = 3.557; p = .031); meaning in life (F = 7. 149; p = .001); search for meaning (F = 6.612; p = .001); proactive coping (F = 8.036; p = .000); avoidance (F = 5.572; p = .005) and self-esteem (F = 17.499; p = .000); social anxiety (F = 2.722; p = 0.49). Supposed difference in the level of depression between the control group and the groups of caregivers was not confirmed. The results of the *post hoc* analyses are described in **Table 3**. Descriptive statistics for the compared groups is presented in **Table A1** in the **Appendix**.

**Table 3:** Significant differences between the control group and caregivers groups.

Dependent variable	(I) code	(J) code	Mean difference = (I-J)	Std. error	Sig.	95% confidence interval	
						Lower bound	Upper bound
Optimism	control group	parent caregiver	.12862	.16238	.429	-.1920	**.4492**
		child caregiver	.42661^*^	.18171	.020	.0678	**.7854**
Positive emotions	control group	parent caregiver	.00750	.13302	.955	-.2551	**.2701**
		child caregiver	.41472^*^	.14885	.006	.1208	**.7086**
	parent caregiver	control group	-.00750	.13302	.955	-.2701	**.2551**
		child caregiver	.40721^*^	.12597	.001	.1585	**.6559**
Negative emotions	control group	parent caregiver	-.29164^*^	.11356	.011	-.5159	**-.0674**
		child caregiver	-.33370^*^	.12708	.009	-.5846	-.0828
Engagement	child caregiver	control group	-.32925^*^	.13031	.012	-.5865	**-.0719**
		parent caregiver	-.30152^*^	.11028	.007	-.5193	**-.0838**
Relationships	control group	parent caregiver	.19137	.14824	.199	-.1013	**.4841**
		child caregiver	.41583^*^	.16589	.013	.0883	**.7434**
Meaning in life	parent caregiver	control group	.14884	.14445	.304	-.1364	**.4341**
		child caregiver	.38017^*^	.13680	.006	.1101	**.6503**
Achievements	child caregiver	control group	-.38239^*^	.11653	.001	-.6125	**-.1523**
		parent caregiver	-.38176^*^	.09862	.000	-.5765	**-.1870**
Physical health assessment	child caregiver	control group	-.47269^*^	.19606	.017	-.8598	**-.0856**
		parent caregiver	-.45705^*^	.16593	.007	-.7847	**-.1294**
Happiness	child caregiver	control group	-.46711^*^	.16967	.007	-.8021	**-.1321**
		parent caregiver	-.39926^*^	.14359	.006	-.6828	**-.1157**
Loneliness	control group	parent caregiver	.35710^*^	.16539	.032	.0305	**.6837**
		child caregiver	.47269^*^	.18508	.012	.1072	**.8381**
Presence of meaning in life	parent caregiver	control group	.20888	.12948	.109	-.0468	**.4645**
		child caregiver	.46161^*^	.12262	.000	.2195	**.7037**
Search for meaning in life	child caregiver	control group	.62977^*^	.19763	.002	.2395	**1.0200**
		parent caregiver	.53521^*^	.16725	.002	.2050	**.8655**
Proactive coping	parent caregiver	control group	.25992^*^	.10971	.019	.0433	**.4766**
		child caregiver	.39975^*^	.10390	.000	.1946	**.6049**
Avoidance	child caregiver	control group	.36919^*^	.17726	.039	.0192	**.7192**
		parent caregiver	.49823^*^	.15001	.001	.2020	**.7944**
Self-esteem	control group	parent caregiver	.19887^*^	.05052	.000	.0991	**.2986**
		child caregiver	.33344^*^	.05653	.000	.2218	**.4451**
	parent caregiver	control group	-.19887^*^	.05052	.000	-.2986	**-.0991**
		child caregiver	.13457^*^	.04784	.006	.0401	**.2290**
Social anxiety	control group	parent caregiver	-.20767	.19385	.286	-.5905	**.1751**
		child caregiver	-50891*	**.21803**	**.021**	**-.9394**	**-.0784**

* denotes significant differences

Bold are the significant differences.

The respondents from the control group revealed significantly higher scores for optimism than the group of children caregivers and experienced significantly fewer negative emotions, as compared to both parents and children caregivers. In the control group, perceived relationships did not differ from those of parents caregivers, but were significantly higher than those of children caregivers. The scores for experienced loneliness were significantly lower in the control group, as compared to the groups of both children and parents caregivers. Sense of accomplishment was significantly lower in the children providing care for a parent with a mental disorder, as compared to the control group. Children caregivers experienced significantly fewer positive emotions than the control group and the parents of children with a mental disorder. Parents caregivers revealed a significantly higher meaning in life, as compared to children caregivers. Avoidance was a significantly more preferred strategy in children caregivers, as compared to the control group and parents caregivers. Experience of happiness rated significantly lower in children caregivers, as compared to the control group and parents caregivers. Children caregivers were significantly more likely to be in the process of search for meaning in life, as compared to the control group and parents caregivers and experience higher social anxiety compared to the control group. Parents caregivers had significantly more proactive coping, as compared to the control group and children caregivers.

### 3.3 Predictors of self-esteem

**Figure 1:**
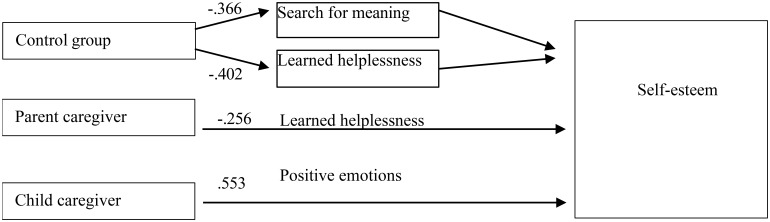
Self-esteem predictors for the control group and the caregivers.

In view to test H2, supposing different pathways, predicting self-esteem for the parents and children caregivers and the control group, a regression analysis of the predictors of self-esteem across the three groups was also carried out, as it is assumed that self-esteem is a characteristics that not only refers to the way in which one perceives oneself, but also to the relatively stable disposition that is developed in early childhood and remains as such later in life. Predictors of self-esteem with an independent effect were different in the three groups compared (**Figure 1**). Regression analyses revealed a model for the control group with an explained variance of 32% (F = 10.146; p = .000; Durbin-Watson 1.645; CI = 95%) and the predictors of self-esteem, low learned helplessness (β = -.402) and search for meaning in life (β = -.366). In the parents of children with a mental disorder, the model accounted for only 0.5% (F = 5.625; p = .020; Durbin-Watson = 2.071) with only one predictor - that of low learned helplessness - with an independent effect (β = -.256; t = -2.372; p = .020). For children caregivers, the only predictor of self-esteem with an independent effect were the positive emotions, explaining 27% of the variance in self-esteem (Adjusted R Square = .267; Durbin-Watson 1.915; F = 17.041; p = .000; β = .533; t = 4.128; p = .000) (**Figure 1**).

### 3.4 Individual effects

As far as H3 is concerned, specificity in the individual effects was observed within each individual group in respect to gender, age, education, and marital status. In all groups individual variables had a partial effect, however the effect of gender differences was large in size, and of marital status – medium to large.

Concerning *gender differences* in the control group women maintained a more optimistic attitude, more engagement and a sense of meaning in life than men. They were more likely to seek emotional support and less likely to use avoidance as a coping strategy. In the group of parents of a child with mental disorder gender differences were observed only in the significantly higher scores for pessimistic attitude and the lower self-esteem in women, as compared to men. The only gender difference in the group of children caring for a parent with a mental disorder was with regard to the search for meaning, which was more pronounced in males than in females. All gender effects were of a large size (**Table 4**).

**Table 4:** Gender differences in the three groups.

	Gender	Mean	Std. deviation	t	Sig.	Cohen’s d pearson’s r
Control group						
optimism	woman	3.8990	.56202	2.532	.016	**d = 1.18** **r = 0.507**
	man	3.2778	.49065			
engagement	woman	3.8182	.54065	2.280	.028	**d = 1.04** **r = 0.463**
	man	3.2778	.49065			
meaning in life	woman	4.0758	.58791	3.209	.003	**d = 1.48** **r = 0.595**
	man	3.2500	.52440			
search for emotional support	woman	3.3576	.59531	2.329	.025	**d = 1.18** **r = 0.507**
	man	2.7667	.38816			
avoidance	woman	2.0505	.72227	8.392	.044	**d = -0.955** **r = -0.431**
	man	2.6667	.55777			
Parents caregivers						
pessimism	woman	2.5524	.82608	2.413	.018	**d = 0.716** **r = 0.337**
	man	1.9167	.94415			
self-esteem	woman	2.9500	.25238	-2.478	.015	**d = -0.817** **r = -0.378**
	man	3.1417	.21515			
Children caregivers						
search for meaning in life	woman	3.4056	.69197	2.429	.019	**d = -1.11** **r = -0.485**
	**man**	**3.9778**	**.23333**			

Bold are higher means.

Concerning *age effects* in the control group and in the group of children caring for a parent with a mental disorder no significant effect was observed. In the group of parents, age only had an effect on perceived achievements - this perception was significantly more expressed in the group of parents under the age of 30 (MD = .56944; p = .006).

In respect to *education effec*t education was only compared in terms of secondary and higher education as there were almost no subjects with primary and college education. Education in the control group had an effect on negative emotions and experience of happiness - respondents with higher education were less likely to experience negative emotions and more likely to experience happiness than those with secondary education. More significant effects of education were observed in the group of caregivers. Parents with higher education reported more optimistic attitude, experienced more positive and less negative emotions, were more engaged, had more satisfying relationships, experienced more achievement and happiness, used preventive coping more often and experienced less learned helplessness and less loneliness, as compared to those with secondary education. In the group of children caregivers of a parent with a mental disorder, education had a significant effect on proactive coping and learned helplessness - graduates were significantly more likely to use proactive coping and were significantly less likely to experience learned helplessness. All differences were of a medium and large effect size (**Table 5**).

**Table 5:** Effect of education.

	Education	N	Mean	Std. Deviation	t	Sig	Cohen's d Pearson's r
Control group							
negative emotions	higher	47	2.6250	.36898	7.852	008	d = -0.886 r = -0.405
	secondary	30	**3.0145**	.50000			
happiness	higher	47	**3.8261**	.57621	4.719	036	d = 0.692 r = 0.327
	secondary	30	3.3750	.71880			
Parents caregivers							
optimism	higher	47	**3.9007**	.61351	3.032	.003	d = 0.673 r = 0.319
	secondary	30	3.3556	.96662			
positive emotions	higher	47	**3.7979**	.53831	2.665	.009	d = 0.609 r = 0.291
	secondary	30	3.4333	65302			
negative emotions	higher	47	3.0496	.57306	-2.146	035	d = -0.504 r = -0.244
	secondary	30	**3.3333**	55364			
engagement	higher	47	**3.8440**	.51476	2.016	047	d = 0.460 r=.637
	secondary	30	3.5778	.63688			
relationships	higher	47	**3.9078**	.70780	2.851	.006	d = 0.653 r = 0.310
	secondary	30	3.4000	.84145			
achievements	higher	47	**3.9362**	.43204	2.166	.034	d = 0.491 r =.567
	secondary	30	3.6889	.56686			
happiness	higher	47	**3.7660**	.63289	2.632	.010	d = 0.599 r = 0.287
	secondary	30	3.3333	.80230			
loneliness	higher	47	**3.7021**	.68888	3.738	.000	d = 0.860 r = 0.395
	secondary	30	3.0667	.78492			
preventive coping	higher	47	**3.6511**	62480	2.826	.006	d = 0.644 r = 0.307
	secondary	30	3.1967	.77792			
learned helplessness	higher	47	2.1837	.55645	-2.430	.018	d = -0.540 r = -0.261
	secondary	30	**2.5772**	.86686			
Children caregivers							
proactive coping	higher	47	**3.6638**	40600	2.851	.006	d = 0.792 r = 0.368
	secondary	30	3.2372	64480			
learned helplessness	higher	47	2.1774	.50486	3.738	.000	d = -1.259 r = -0.533
	secondary	30	**2.8026**	.48815			

Bold are higher means.

The only significant *marital status effect* observed in the control group was in physical health assessment (F = 4.338; p = .0110), which scored higher in married people (M = 4.011), as compared to those living in cohabitation (M = 3.2000). Among the parents caregivers married people had more satisfactory relationships than single ones (MD = 0.66667; p = 0.011). In comparison to single people, married people reported higher scores for sense of meaning in life and physical health (MD = .65000; p = .010 and MD = .97500; p = .001, respectively), they experienced more frequently happiness (MD = .70000; p = .008) and less frequently loneliness (MD = .68750; p = .020). They were less likely to be in the process of search for meaning in life (MD = -.46250; p = .043). Among the children caring for a parent with a mental disorder, the married ones also had better relationships than single ones (MD = .66667; p = .011); married people also revealed higher values for meaning in life than single people (MD = .65000; p = .010).

Family anamnesis accounted significant difference with large effect for two coping strategies – search for instrumental and search for emotional support between respondents with and without history of mental disorder in the family (**Table 6**). Respondents with family anamnesis are much prone to search for help.

**Table 6:** Differences between the groups of respondents with and without history of mental disorder in the family.

	Family anamnesis	N	Mean	Std. deviation	t	Sig. (2-tailed)	Cohen’s d pearson’s r
Search for instrumental support	Yes	40	3.6906	.62467	2.894	.004	**d = .563** **r = .271**
	No	87	3.3204	.68907			
Search for emotional support	Yes	40	3.7000	.67482	2.721	.007	**d = .533** **r = .258**
	No	87	3.3126	.77502			

Bold are higher means.

For parents the diagnosis of the child and age of the child had not accounted significant effect. For children caregivers there is no difference the diagnosis of the parent they care for neither is the parent mother or father. What has specific effect for them is the position of the child in the family – the birth order – is the child first, second (third) or only child. The only children had higher levels of depression compared to those having siblings – first (mean difference =. 37115*; p = .026; CI = 95%) and second child in the family (mean difference =. 33615*; p = .036; CI = 95%). *Post hoc* analyses revealed that the first child in the family reported higher achievements compared to the sole children (mean difference.50427; p = .023; CI = 95%), engagement (mean difference = .61966*; p = .013; CI = 95%), and perceive more meaning in life compared to sole children (mean difference =. 67607*; p = .003; CI = 95%) and second children in family (mean difference =. 68222*; p = .010; CI = 95%).

## 4 Discussion

In mental disorders, there is a genetic component that can lead to experience of guilt (59). Studies on families with a member with schizophrenia face a number of challenges, as this group of patients has a relatively low reproductive rate (60). The incidence of schizophrenia among first-degree relatives is between 10 and 15% (61). Caregivers of mentally ill tend to blame someone or something (62). Family members feel guilty about their relative’s illness, so they often avoid various social situations (63). The present study involved 10 children caregivers of parents diagnosed with schizophrenia (6 mothers and 4 fathers), and only 3 of them had children. Other research has also confirmed that caring for a mentally ill person has significant negative impact on the family, and the lack of support leads to deterioration in their health and well-being, social functioning and financial stability (64), which was also confirmed by the results of our study. Providing care for a mentally ill person leads to stress and deterioration in relationships, in addition to affecting the caregiver’s physical and mental health (65), and our results also revealed the negative impact of caring on the caregiver’s relationships, physical health, and well-being; however, and difference in social anxiety was observed between the children caregivers and the control group.

The results of an European study have also confirmed that providing care for people with mental disorders, which is predominantly carried out by family members, is accompanied by an enormous burden and has a negative impact on the health of caregivers (66). Caregivers experience anxiety, which consists in experiencing helplessness most frequently caused by the lack of knowledge how to provide care for their loved ones with a mental disorder (2). Results of this study partially replicated the higher social anxiety, experienced by children caregivers compared to the control group. Learned helplessness, however is not reported in this study. It is not expressed neither among caregivers, nor among the respondents from the control group.

Concerning parental role parents having a child with mental problems often experience them as a loss, experiencing constant fear and anxiety about their child’s future (67). This anxiety about the future results from the child’s need of long-term training by specialists, as well as support during their education. The mothers’ anxiety is also related to the long-term effects of mental illness on their children (68). Mothers experience fear, frustration and guilt that are more marked when they have difficulty coping with their child’s behavior, and because of their own attitude towards the child (69). In a study of 304 parents of children with ASD, mothers predominated as caregivers (59.5%), and the most frequently used strategy was seeking social support, while avoidance was least frequently used (70). The overall care of children with ASD is taken by mothers, so they are highly involved in caregiving and bear the burden entailed by this care (71). Seeking social support is one of the most common and effective strategies used by parents of children with ASD (72). Other authors have also confirmed that mothers are more likely to seek social support (from family members, friends and professionals), as compared to fathers (73). There is debate as to whether avoidance represents a positive or negative coping mechanism for parents of children with ASD. Some have suggested that avoidance may have a negative impact on mental health, while others suggest that it may temporarily reduce the impact of stressors on mental health (74). For parents of children with ASD, the strategy of avoidance produces short-term results; however, it is not appropriate for chronic stressful situations (75), as avoidance may lead to increased anxiety and depressive symptoms in the long term (76). Mothers more often seek social and emotional support, whereas fathers more often avoid this. These differences have been explained with the fathers’ employment, due to which they spend more time outside the home and family (77). Raising a child with a mental disorder significantly affects the mental health and resources of the parents, resulting in decreased psychological well-being (78). The results obtained in our study replicated the more negative situation of such parents, however they demonstrated more proactive attitude and coping in this group of respondents, especially those with higher education. The results of this study did not report social anxiety, search for help or avoidance among parents of children with mental disorder. On the contrary, they revealed a clear orientation to proactive performance as preferred coping strategy.

As expected, the control group had better scores than the caregivers. High proactive coping was specific for parents caring for a child with a mental disorder, revealing it to be a beneficial strategy in this situation. Although there was no difference in depression between the caregivers and the control group, the caregivers scored higher for experienced loneliness, which is considered to be a risk factor for depression (55). No significant differences were found for learned helplessness, which was relatively mildly expressed in all respondent groups.

The above-mentioned findings were consistent with the evidence reported by some authors that self-esteem, social support and life satisfaction were lower in parents of children with ASD, as compared to parents of healthy children (53). Other researchers have suggested that self- esteem may act as a buffer against stress - low self-esteem increases a person’s vulnerability to stressful stimuli; in their opinion, self-esteem mediates the relationship between the parenting style and children’s mental health (54). The results outlined for self-esteem of caregivers are thus important, highlighting the specific support needed for parents and children caring for a family member with mental disorder. A number of researchers have pointed out that family adversity influences mental health, with the unfavorable family environments influencing children’s mental health (84). Children with a mentally ill parent are at a higher risk of developing depression and anxiety disorders, as compared to their peers, as well as experiencing a range of difficulties in their studies, including dropping out of school (85, 86). The results of the present study showed difference in social anxiety between the control group and children caregivers and no differences in depression between the control group and the groups of both parents and children caregivers - further research is needed in this area.

Concerning parental role of caregiver in our study, 20 mothers with a mental disorder were included in the group of parents caring for mentally ill children: 13 mothers had depression (7 of which had postpartum depression that triggered off depression later in life) and 7 mothers had panic disorder. Their average age was 39.9 years, with an average age of giving birth 33.14 years. As a whole, the average age of parents caring for mentally ill children in the present study was the highest, as compared to the other two groups studied, reflecting the current trend of delaying parenthood. Women frequently have aspirations for higher education, so they become mothers and take on different social roles and responsibilities with regard to others at a slightly later age.

Some researchers have found the prevalence of postpartum mental disorders to be 20.42%, with postpartum depression being the most commonly diagnosed at 17.96%, anxiety disorders at 11.97%, obsessive-compulsive disorder at 4.5% and posttraumatic stress disorder at 1.41%; comorbidity among the various mental disorders has been found to be high - around 58.62% (79). In our study the 7 mothers with postpartum depression developed depression later in life, while the other 13 mothers were diagnosed with mental disorder in the course of their child’s atypical development – the period from the initial manifestations of the child’s mental disorder to the establishment of the diagnosis coincided with the period of development of the mother’s mental disorder.

Achieving control over the disease symptoms is essential, since the presence of a mental disorder in the mother can bring about disturbance in the child’s development, influence the child’s emotional regulation and social functioning starting at an early age (80, 81), as well as increase the risk of developing a mental disorder in adolescence (82). Other researchers have also supported the belief that a mother’s postpartum depression influences her child’s development, so they have suggested the hypothesis of intergenerational transmission of depression from mothers to their children (83).

Parents of a child with mental disorder had more pronounced proactive coping than the children of a parent with mental disorder and the control group. They showed no difference in optimism and experienced positive emotions, as compared to the control group; they evaluated their relationships in the same way as the control group respondents, and like them, had a sense of meaning in life, although they experienced more negative emotions and feelings of loneliness and revealed lower self-esteem, as compared to the control group. Main predictor of their self- esteem was the low learned helplessness.

Parents of children with a mental disorder adapted mainly through proactive coping. Clearly the group of children caregivers of a parent with a mental disorder was the most vulnerable and most frequently women were the caregivers, having more difficulties in adapting to this role, as compared to men. Children caregivers had less hope and motivation to change the situation, they had the lowest scores for self-esteem, meaning in life and happiness, as well as accomplishment and experience of positive emotions. They used avoidant coping and showed search for meaning in life more frequently than the other two groups as a way of escaping from reality.

Concerning children caregivers in this study, 12 children with mental disorder (10 with depression and 2 with panic disorder) were caring for 10 mothers and 2 fathers with a mental disorder. The average age at which their parents were diagnosed with the mental disorder was 25.25 years. Our study on daughters caring for their mentally ill mothers found that 10 daughters with a mental disorder were caring for their ill mothers (5 mothers had bipolar disorder, 4 had depression and 1 had schizophrenia). Among the daughters providing care 8 were diagnosed with depression and 4 with panic disorder; the average age at which diagnosis was established was 23 years. Six of the daughters caring for their mentally ill mothers were unmarried and five had no children. The results of the present study also confirmed presence of mechanisms associated with illness transmission from mothers to daughters; however, because of their relatively small number no specific conclusions can be made.

Children caring for a parent with a mental disorder had lower optimistic attitudes, as compared to the control group and the group of parents caregivers; they scored lowest for experienced positive emotions, sense of achievement and engagement, as well as physical health assessment, experienced happiness and meaning in life; scored highest for search for meaning in life, were most likely to use avoidant coping and maintained lowest self-esteem. They scored more negative emotions, higher social anxiety, more distressed relationships, and less low proactive coping than the control group. For them, the main predictor of self-esteem were positive emotions.

The group of children of a parent with a mental disorder appeared to be the most vulnerable, which, confirmed the deficit on the one hand, and the social isolation, financial and social difficulties, on the other. All this highlighted the group of children caregivers as the one most in need of additional support.

All gender effects are large and indicate better self-regulation for women than for men in the control group and, on the contrary, significantly worse adjustment in the caring role than for men.

The specific role of women in caregiving has been outlined in a number of previous publications. In contrast to the results obtained in the control group, where women were more optimistic than men, mothers of children with a mental disorder were more pessimistic and had lower self-esteem than men. Women caring for a parent with a mental disorder were less likely to search for meaning in life, but were as likely to have meaning in life as the women in the control group. All gender effects had large size effect and indicated better self-regulation of women than men in the control group and, on the other hand, significantly worse adjustment to their caregiving role, as compared to men.

The present study revealed that education is one of the significant factors influencing the variables analyzed. When comparing respondents according to their education level, significant differences were also found, and these were specific to each group. In the control group, higher education was associated with less negative emotions and more happiness. For caregivers, higher education was associated with more proactive orientation and less learned helplessness. The largest number of education effects were reported for parents caring for a child with a mental disorder: higher education contributed to greater optimism, more positive emotions, fewer negative emotions, more engagement, relatedness, achievement, experience of more happiness and less loneliness, more proactive coping and less learned helplessness, as compared to parents with secondary education. These results replicated that the less educated people have poorer quality of life, physical health and social functioning (56). Higher levels of education provide more opportunities and knowledge to cope with stressful situations, leading to a better quality of life. The more educated people have better jobs and income, more resources, enabling them to provide better care and quality of life for their loved ones with a mental disorder (57). In our study, the greater part of the respondents had higher education and were employed. Several authors have analyzed the relationship between employment and quality of life for caregivers of mental patients. It has been suggested that work provides opportunities to build a network for socializing with others, which may help to reduce emotional distress. Our results and the reports of others studies (87, 88) confirmed the fact that higher level of education and employment result in better coping with care provision. In addition, employment provides income and reduces the financial difficulties that caregivers may experience (58). Marriage has been shown to be supportive in the caregivers groups.

Single respondents had lowest scores for positive emotions, engagement, favorable relationships, meaning in life, achievement and experienced happiness; on the other hand, they rated highest regarding the search for meaning in life, used proactive coping more rarely and had the lowest self-esteem.

H1 was partially confirmed – there are specific differences between the control group and the two groups of caregivers for psychiatric family member. Important result are the differences, accounted for the adjustment of parents and children. The results confirmed our expectations of a relatively positive picture of the respondents’ well-being, a partial effect of the individual variables, a large-size gender effect and high specificity in personality disposition, preferred coping strategies and life orientation in the control group and the caregivers of a family member with mental disorder. An important point was the absence of a difference in the depression and social anxiety scores between the control group and the parents of a child with mental disorder, which confirms the universal characteristics of self-regulation and adaptation to situations. However, this did not diminish the specificity in the caregivers groups and highlighted significant areas in which they needed support.

In confirmation of H2 different predictors are reported for the predictors of self-esteem. Self-esteem is predicted by low levels of search for meaning in line and learned helplessness for the control group as independent predictors. For the parents caregivers self-esteem is predicted only by low level of learned helplessness. For children caregivers self-esteem is predicted only by high level of experienced positive emotions. This reveals very different basic needs of the two groups of caregivers – parents need mainly support not to feel helplessness but children need support in view to have more positive emotions.

H3 was also confirmed – individual effects accounted depending on gender, age, education, and marital status indicate different patterns of the effects for the control group and the two groups of caregivers. For the control group there are most gender differences, revealing that men are less avoidant and that women are more optimistic, engaged, and have both higher meaning in life and search for meaning. For parents caregivers women have lower self-esteem and higher pessimism compared to men. And for children caregivers women have more expressed search for meaning in life. Education has also rather specific effect for each of the three groups – as far as for the control group higher education leads to less negative emotions and more happiness, for children caregivers it is related to more proactive coping and less learned helplessness. Most effects education has for parents caregivers - higher education is related to optimistic attitude, more positive and less negative emotions, more engagement, better relationships, sense of achievements, perceived happiness and less loneliness and learned helplessness and more preventive coping. Marital status has only one significant effect in the control group – married people score their physical health higher compared to those, living in cohabitation. More effects are observed among parents caregivers - married people had more satisfactory relationships than single ones and those, living in cohabitation, report higher meaning in life and physical health, experience more frequently happiness and less frequently loneliness and rarely are in the process of search for meaning in life. Among the children caring for a parent with a mental disorder, the married report more satisfaction with relationships than single ones, living in cohabitation, and divorced and find more meaning in life compared to single.

These results lead to the conclusion that irrespective the life situation, adjustment and self-regulation can be effectively maintained, however there are clear differences between the healthy respondents with healthy children and parents and the groups of caregivers for a family member with psychiatric disorder. Following the guidelines for support of families, the outlined patterns of needs can facilitate consulting and treatment.

## 5 Limitations and contributions of the study and future research

The main limitation of the study is the convenient sample and the small number of respondents in each subgroup. Further research should be conducted on the impact of the diagnosis of the mentally ill family member, the child’s birth order and the effect of the onset of illness in the parent for children caregivers, the transmission of mental diseases from parent to child, and an in-depth analysis of the impact on the family situation should be performed. Better differentiation and profiling depending on the diagnosis of the family member, the position of the caregiver – parent or child, the age of the caregiver when the diagnosis had been determined, the mental state of the caregiver, as this will contribute to outlining the different profiles and need of information and support for the family and effective family functioning.

The position of parent or child caring for family member with mental disorder highlight different coping strategies, perceived components of well-being, and life orientation are different from those observed in healthy families, and our study revealed the different patterns of adjustment and self-regulation. Furthermore, the specific predictors of self-esteem are also a contribution to understanding the specific situation along with the different patterns of individual effects for each group of caregivers. The lack of difference in depression between the control group and caregivers of a family member with mental disorder needs future in-depth analysis in view to robust conclusions. We consider these results preliminary findings, supporting the need of further study of the different life patterns of the caregivers. Sampling and small groups sizes do not allow unambiguous differentiated interpretation and generalizability of the results. Data collection will allow better differentiation of the effect of the diagnoses, mental health status of caregivers and the effect of other variables both as independent antecedents of well-being and as interrelated variables.

In future research focus will be given also to different instruments, which will help for outlining the experienced anxiety, depression and life dispositions of caregivers compared to the control group.

The contribution of the study, however, is in outlining various patterns of family caregiving. Parents caring for a child with mental disorder have proactive behavior, as well as meaning in life and relationships. Children caring for a parent with a mental disorder lack meaning in life, which makes them more avoidant, pessimistic, searching for meaning in life and in need of more positive emotions. The different patterns of caregiving suggest the need of future research and outline the following key points for support: keeping the parents caregivers informed about the resources available in the community and facilitating their proactive attitudes; promoting the sense of meaning in life, positive emotions and more effective coping in children caregivers. Mothers most frequently take the responsibilities of providing care, are more pessimistic and have lower self-esteem, as compared to fathers caregivers. The children caring for a parent with a mental disorder are more likely to search for meaning in life. For them the position of the child in the family – only child and birth order has also specific effect. The conclusions drawn have practical relevance, and can be used to guide medical practitioners, outlining a specific profile of the care providers.

A specific focus for future research are mothers developing symptoms of mental disorder after delivery (postpartum depression), followed by mental disorder development in the course of their child’s diagnosing with mental disorder - they revealed a different pattern of adjustment and self-regulation. This opens up new research areas that would contribute to understanding the experience of women who take the burden of providing care and cope with the new situation as a long-term perspective. Such mothers should be referred for early intervention, so that their developing a mental disorder can be prevented. On the other hand, the children of mentally ill parent is undoubtedly the most vulnerable group, which need preventive and continuous support. The different patterns highlighted in this study suggest further research for in-depth analysis of the caregivers profile and support depending on their life adjustment and performance in view to promotion both their individual and family well-being.

## Data Availability

The raw data supporting the conclusions of this article will be made available by the authors, without undue reservation.

## Appendix

**Table A1:** Descriptive statistics for the control group and the two groups of caregivers.

		N	Mean	Std. deviation	Std. error mean
Optimism	control group	39	3.8034	.59095	.09463
	parent caregiver	82	3.6748	.85101	.09398
	child caregiver	45	**3.4222**	.93041	.13870
Pessimism	control group	39	2.2564	.78532	.12575
	parent caregiver	82	2.4593	.86803	.09586
	child caregiver	45	2.4593	.81739	.12185
Positive emotions	control group	39	3.6538	.55196	.08838
	parent caregiver	82	3.6463	.65493	.07233
	child caregiver	45	**3.2667**	.80904	.12060
Negative emotions	control group	39	2.8547	.46387	.07428
	parent caregiver	82	**3.1463**	.60200	.06648
	child caregiver	45	**3.1556**	.60553	.09027
Engagement	control group	39	3.7350	.56288	.09013
	parent caregiver	82	3.7073	.58885	.06503
	child caregiver	45	**3.4074**	.65091	.09703
Relationships	control group	39	3.9231	.56951	.09119
	parent caregiver	82	3.7317	.78294	.08646
	child caregiver	45	**3.5556**	.80403	.11986
Meaning in life	control group	39	3.9487	.64680	.10357
	parent caregiver	82	4.0976	.76352	.08432
	child caregiver	45	3.7556	.74332	.11081
Achievements	control group	39	3.8462	.47045	.07533
	parent caregiver	82	3.8455	.51903	.05732
	child caregiver	45	**3.4963**	.57570	.08582
Physical health assessment	control group	39	3.8205	.79046	.12658
	parent caregiver	82	3.8049	.88106	.09730
	child caregiver	45	**3.4000**	.96295	.14355
Happiness	control group	39	3.6410	.66835	.10702
	parent caregiver	82	3.5732	.77029	.08506
	child caregiver	45	**3.2222**	.82266	.12263
Loneliness	control group	39	3.8205	.88472	.14167
	parent caregiver	82	3.4634	.80423	.08881
	child caregiver	45	**3.3778**	.88649	.13215
Meaning in life	control group	39	3.7179	.59421	.09515
	parent caregiver	82	3.9268	.72522	.08009
	child caregiver	45	3.4800	.60663	.09043
Search for meaning in life	control group	39	2.8615	.91324	.14624
	parent caregiver	82	2.9561	1.00764	.11127
	child caregiver	45	**3.5200**	.66661	.09937
Proactive coping	control group	39	3.5562	.62354	.09985
	parent caregiver	82	**3.8161**	.48406	.05346
	child caregiver	45	3.4581	.58028	.08650
Reflexive coping	control group	39	3.4755	.62201	.09960
	parent caregiver	82	3.5987	.63031	.06961
	child caregiver	45	3.3515	.64358	.09594
Strategic planning	control group	39	3.1923	.86310	.13821
	parent caregiver	82	3.4360	.89994	.09938
	child caregiver	45	3.1556	.85162	.12695
Preventive coping	control group	39	3.3538	.65528	.10493
	parent caregiver	82	3.4951	.70692	.07807
	child caregiver	45	3.3022	.42932	.06400
Search for instrumental support	control group	39	3.1827	.74220	.11885
	parent caregiver	82	3.4741	.73801	.08150
	child caregiver	45	3.3694	.59110	.08812
Search for emotional support	control group	39	3.2667	.60408	.09673
	parent caregiver	82	3.5098	.76022	.08395
	child caregiver	45	3.2978	.75993	.11328
Avoidance	control group	39	2.1453	.72867	.11668
	parent caregiver	82	2.0163	.87159	.09625
	child caregiver	45	**2.4889**	.76409	.11390
Learned helplessness	control group	39	2.2227	.53626	.08587
	parent caregiver	82	2.3376	.70448	.07780
	child caregiver	45	2.4784	.69224	.10319
Self-esteem	control group	39	3.1769	.31327	.05016
	parent caregiver	82	**2.9780**	.25533	.02820
	child caregiver	45	**2.8422**	.21584	.03218
Social anxiety	control group	39	2.2784	1.02333	.16386
	parent caregiver	82	2.4861	.95551	.10552
	child caregiver	45	**2.7873**	1.04607	.15594
Depression	control group	39	1.9897	.40102	.06421
	parent caregiver	82	2.1366	.35154	.03882
	child caregiver	45	2.1222	.44307	.06605

Bold are higher means.
